# Methodological Assessment of High-Throughput Sequencing Platforms: Illumina vs. MGI in Clinical-Grade *CFTR* Genotyping

**DOI:** 10.3390/ijms262311701

**Published:** 2025-12-03

**Authors:** Marianna Beggio, Edoardo Peroni, Eliana Greco, Giulia Favretto, Dario Degiorgio, Antonio Rosato, Mosè Favarato

**Affiliations:** 1 UOSD Genetica e Citogenetica, Dipartimento di Direzione Medica del Presidio Ospedaliero di Mestre, AULSS3 Serenissima, 30172 Venezia, Italy; 2 Immunology and Molecular Oncology Unit, Veneto Institute of Oncology, IOV-IRCCS, 35128 Padova, Italy; 3 Department of Surgery Oncology and Gastroenterology, University of Padova, 35122 Padova, Italy

**Keywords:** high-throughput sequencing, NGS, MGI, Illumina, *CFTR*, cancer

## Abstract

The growing demand for precision diagnostics in cystic fibrosis and other genetic disorders, such as cancers, is driving the need for sequencing platforms that combine analytical robustness, scalability, and cost-efficiency. In this study, we performed a direct comparison between two leading Next-Generation Sequencing (NGS) platforms, MiSeq (Illumina, CA, USA) and DNBSEQ-G99RS (MGI Tech Co., Shenzhen, China), using a CE-IVD-certified *CFTR* panel (Devyser AB), selected for its complexity and variant spectrum, including SNVs, CNVs, and intronic polymorphisms. A total of 47 genomic DNA samples from routine clinical activity were analyzed on both platforms. Illumina sequencing covered all *CFTR* variants using standard workflows, while MGI data were generated from residual diagnostic DNA, with informed consent. Sequencing data were processed using Amplicon Suite v3.7.0 for variant calling, annotation, and ACMG classification. Quality control metrics and platform-specific parameters were also evaluated. Both platforms demonstrated complete concordance in variant detection, including SNVs, CNVs, and complex alleles (e.g., Poly-T/TG). Illumina exhibited slightly superior basecalling quality and allelic frequency uniformity, while MGI achieved higher sequencing depth (mean ~2793×) and demultiplexing efficiency. No false positives, false negatives, or discordant HGVS annotations were observed. The use of full-gene *CFTR* sequencing enabled granular and technically rigorous cross-platform validation. These findings confirm the analytical equivalence of Illumina and MGI for diagnostic genotyping. Moreover, MGI’s greater data output and flow cell capacity may offer tangible advantages in high-throughput settings, including somatic applications such as liquid biopsy and molecular oncology workflows.

## 1. Introduction

### 1.1. Cystic Fibrosis: Clinical Relevance and Genetic Basis

Cystic Fibrosis (CF) is a multisystem, autosomal recessive disorder that predominantly affects the respiratory and digestive systems. It is considered the most common life-threatening genetic disease among individuals of European ancestry, with an incidence rate ranging between 1 in 2500 and 1 in 3500 live births in Europe and North America [1]. The disease is caused by inherited mutations in the *CFTR* gene, which encodes a chloride channel protein responsible for maintaining fluid homeostasis across epithelial membranes [2,3].

Clinically, CF is characterized by chronic pulmonary infections, progressive bronchiectasis, pancreatic insufficiency, and elevated levels of chloride in sweat. Many patients also suffer from male infertility, specifically due to congenital bilateral absence of the vas deferens (CBAVD), a hallmark of CF-related disorders [4]. Advances in clinical care and the introduction of CFTR modulator therapies have significantly improved life expectancy; however, early and accurate molecular diagnosis remains critical for guiding therapeutic decisions, genetic counseling, and family planning [5].

### 1.2. The CFTR Gene and Its Variant Landscape

The *CFTR* gene (Cystic Fibrosis Transmembrane Conductance Regulator), located on chromosome 7q31.2, spans over 190 kilobases and includes 27 exons. It encodes a 1480 amino acid protein that belongs to the ATP-binding cassette (ABC) transporter family [6]. The CFTR protein functions as a cAMP-regulated chloride channel at the apical membrane of epithelial cells, playing a central role in regulating salt and water transport in tissues such as the lungs, pancreas, intestine, and reproductive tract [7].

Since its discovery in 1989 [8], over 2000 sequence variants have been identified in *CFTR*, many of which exhibit complex and context dependent pathogenicity. These variants include single nucleotide variants (SNVs), **s**mall insertions and deletions (indels), **e**xon-spanning copy number variations (CNVs), **d**eep intronic mutations affecting splicing and **p**olymorphisms in regulatory regions (e.g., poly-T and TG repeats)

This genetic heterogeneity poses significant diagnostic challenges, necessitating high-throughput, highly sensitive methodologies capable of detecting both common and rare variants across the entire gene and its regulatory elements. In particular, the ability to detect CNVs and mutations in intronic “hotspots” such as introns 9, 11, and 22 is vital to ensure comprehensive genotyping [9].

### 1.3. Next-Generation Sequencing Technologies: Illumina and MGI Compared

The emergence of high-throughput sequencing technologies, broadly categorized as Next-Generation Sequencing (NGS), has revolutionized the field of genomics by enabling massively parallel sequencing of millions of DNA fragments in a single experiment at affordable cost. Unlike traditional Sanger sequencing, which is limited by throughput and scalability, NGS provides a platform for comprehensive, high resolution, and cost-effective genotyping, essential for both monogenic and polygenic disorders [10,11].

NGS platforms achieve this by fragmenting the genome and sequencing multiple fragments in parallel, allowing for detection of a broad spectrum of SNVs, indels, CNVs, and structural rearrangements. The depth and breadth of coverage afforded by these technologies have made them indispensable in both research and clinical settings, particularly in the context of genetically heterogeneous conditions such as CF [12,13].

In recent years, the increasing availability of targeted gene panels, whole exome sequencing (WES), and whole genome sequencing (WGS) has further enhanced the applicability of NGS in diagnostics, enabling genotype driven personalized medicine and early therapeutic intervention [14,15]. Among the commercial platforms leading this technological advancement, Illumina and MGI Tech Co. have emerged as prominent players, offering distinct yet competitive sequencing solutions based on different underlying chemistries and workflows.

**Illumina** NGS platforms are currently the most widely adopted technologies in clinical and research settings. The core chemistry used by Illumina, known as sequencing-by-synthesis (SBS) chemistry, involves fragmenting DNA, ligating adapters, clonal amplification on a flow cell via bridge amplification, and cyclic reversible terminator-based sequencing [16,17].

Each sequencing cycle involves the incorporation of fluorescently labeled nucleotides, with real time imaging used to determine base identity. This results in highly accurate short read sequences, typically in configurations such as paired end 2 × 150 bp, which are optimal for small to mid-sized targeted panels like *CFTR*. Platforms such as MiSeq, MiniSeq, NextSeq up to NovaSeq offer varying throughput levels, with MiSeq commonly used in diagnostics due to its modest output (1–15 Gb) and relatively short run time [18].

Illumina systems are supported by a mature ecosystem of sample prep kits, base-calling software, and third-party bioinformatics tools, making them an industry standard in clinical genomics.

**MGI’s DNBSEQ** and cPAS Technology powered by MGI Tech Co., a subsidiary of BGI Group, offers an alternative to Illumina with its proprietary DNBSE platform, which combines DNA nanoball (DNB) technology with combinatorial Probe-Anchor Synthesis (cPAS). Unlike Illumina’s bridge amplification, DNBSEQ technology utilizes rolling circle amplification to generate highly uniform DNBs from circularized DNA molecules. These DNBs are then immobilized on patterned arrays for sequencing [19].

Sequencing is achieved through stepwise hybridization and ligation-based incorporation of labeled probes, offering reduced error rates and low levels of index hopping. The DNBSEQ-G99RS instrument is particularly suited for targeted sequencing applications, providing high data output (24–48 Gb per run) with paired-end 150 bp reads, and faster run times due to its high-density patterned arrays.

One of the challenges of using MGI platforms in established Illumina workflows has been library compatibility. However, this has been mitigated by the development of library conversion kits that allow Illumina-prepared libraries (e.g., Devyser CFTR) to be processed on DNBSEQ platforms, thereby promoting interoperability without compromising sequencing quality [20].

As NGS technologies continue to evolve, the comparative evaluation of different platforms has become increasingly essential to inform decisions regarding clinical adoption. Factors such as cost effectiveness, sequencing accuracy, compatibility with third-party assay kits, and seamless integration into existing bioinformatics pipelines must all be considered when selecting a platform for routine diagnostics.

In the field of CF, where *CFTR* genotyping plays a pivotal role not only in confirming diagnosis but also in guiding therapeutic decisions, especially with the emergence of mutation-specific CFTR modulators, accurate and scalable sequencing solutions are of paramount importance. Ensuring consistency in variant detection, adequate coverage across clinically relevant regions, and robustness in polymorphic loci such as Poly-T/TG tracts are critical to achieving diagnostic reliability and therapeutic relevance [21].

This study presents a head-to-head comparison of the MiSeq (Illumina, CA, USA) and DNBSEQ-G99RS (MGI Tech Co. Shenzhen, China) platforms using the CE-IVD Devyser Diagnostic CFTR NGS panel. By evaluating multiple performance dimensions, including library preparation efficiency, sequencing depth, variant call concordance, and downstream informatic compatibility, this work aims to provide evidence-based guidance on platform interchangeability and optimization in the context of *CFTR* molecular diagnostics.

## 2. Results

The expanding landscape of NGS technologies offers several platforms optimized for different performance and cost profiles. Illumina’s MiSeq, a widely adopted benchmark in clinical diagnostics, delivers consistently high basecall accuracy and reproducibility in targeted assays, particularly for gene panels of moderate size [18]. In parallel, MGI’s DNBSEQ technology, including the G99RS system, is emerging as a promising alternative due to its high throughput, reduced cost per base, and compatibility with established library preparation workflows [19,22].

Although early comparative studies have demonstrated analytical consistency across platforms [23,24], platform interchangeability in a clinical context remains underexplored, particularly for complex genomic targets such as the *CFTR* gene, which harbors SNVs, CNVs, and poly-T/TG tract variations with known phenotypic relevance.

Here, we present a systematic head-to-head evaluation of Illumina MiSeq and MGI DNBSEQ-G99RS platforms using a harmonized clinical-grade workflow for *CFTR* genotyping. All upstream processes (DNA extraction, library prep, cleanup) and downstream analyses (FASTQ processing, variant calling via Amplicon Suite) were standardized to minimize pre-analytical bias.

Our comparative framework encompassed (i) read-level metrics: Q30, phasing, error rates, and alignment efficiency; (ii) coverage uniformity across all targeted amplicons; (iii) variant detection concordance for SNVs, CNVs, and poly-T/TG alleles; and (iv) platform-specific efficiency indicators such as chip productivity and recovery consistency.

This analytical comparison aims to assess the clinical equivalence and diagnostic robustness of the two platforms and to guide laboratories in adopting evidence-based criteria for platform selection and validation in precision medicine workflows.

### 2.1. Amplicon-Level Coverage Comparison: Illumina vs. MGI

The analysis of mean coverage per amplicon across the entire *CFTR* panel reveals a consistent advantage of the MGI DNBSEQ-G99RS platform over Illumina MiSeq. Specifically, MGI achieves higher average read depth for nearly every amplicon analyzed. This difference is especially notable for specific target regions such as *CFTR_ex01_02*, *CFTR_ex06_01*, and others, where MGI coverage levels exceed those of Illumina by several fold.

This trend corroborates previously published comparative studies, which have shown that MGI systems tend to deliver more uniform and deeper coverage across targeted regions [23,24,25]. Enhanced coverage depth is a critical parameter in diagnostic sequencing, as it improves the sensitivity of variant detection, minimizes the risk of missing low frequency alleles, and ensures robust representation of all clinically significant loci.

**Figure 1:**
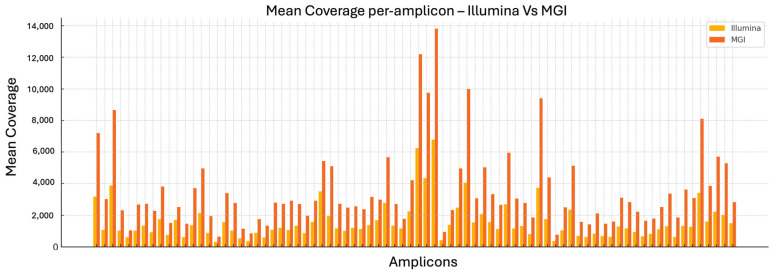
Comparative mean coverage per amplicon obtained using Illumina MiSeq and MGI DNBSEQ-G99RS platforms for the Devyser CFTR NGS panel. Each bar represents the average coverage achieved across 47 clinical samples for a specific *CFTR* target region. MGI consistently demonstrates higher mean coverage values across the majority of amplicons, indicating a more efficient sequencing depth distribution across the panel. Despite these differences, both platforms surpassed the minimum required coverage threshold of 100× for all targets, ensuring robust variant detection. These results support the analytical suitability of both platforms, with MGI showing a potential advantage in terms of per-amplicon read depth.

Overall, the observed increase in per-amplicon mean coverage (Figure 1) with MGI supports its suitability for highly sensitive assays, particularly in diagnostic contexts where comprehensive and uniform coverage is essential.

### 2.2. Distribution of Relative Coverage *Δ* (MGI vs. Illumina)

**Figure 2:**
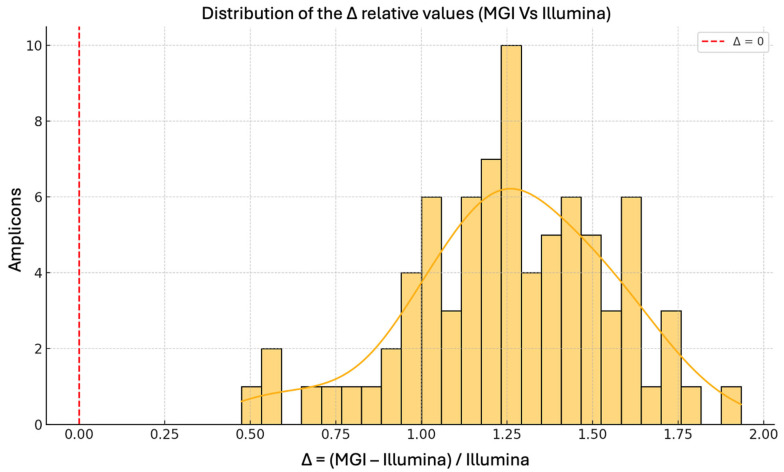
Distribution of Relative Coverage Increase (MGI vs. Illumina) Histogram representing the distribution of the relative difference in mean amplicon coverage between MGI and Illumina platforms, calculated as Δ = (MGI/Illumina) − 1. All values of Δ are greater than zero, indicating that MGI consistently achieved higher coverage across all amplicons analyzed. The majority of Δ values fall between +0.3 and +1.5, corresponding to a 30% to 150% increase in coverage. Several amplicons exceed a Δ of 2, where MGI delivered more than twice the depth obtained with Illumina. These results highlight the superior sequencing efficiency of MGI in terms of per-amplicon read depth.

The histogram in Figure 2 illustrates the distribution of the relative change in average coverage per amplicon between MGI and Illumina platforms, calculated as Δ = (MGI/Illumina) − 1. All Δ values are positive, confirming that MGI consistently outperformed Illumina in terms of sequencing depth across every amplicon within the CFTR panel.

The distribution is right-skewed, with the majority of values concentrated in the range of +0.3 to +1.5. This corresponds to an increase in mean amplicon coverage of approximately 30% to 150% using the MGI platform. Notably, several outliers exceed a Δ of 2.0, indicating that MGI achieved three times higher coverage than the coverage observed with Illumina for certain targets.

This uniform enhancement across all target regions further substantiates the improved depth performance of MGI and aligns with findings from previous comparative studies reporting improved coverage metrics for MGI platforms in amplicon-based sequencing protocols [26]. Such elevated coverage levels are critical for minimizing the risk of allelic dropout and enhancing detection of low frequency variants, especially in clinical diagnostic settings where comprehensive coverage is paramount [27,28].

### 2.3. Coverage Uniformity and Critical Threshold Evaluation

In addition to evaluating overall coverage performance, we assessed the uniformity of sequencing depth across all targeted amplicons by analyzing the standard deviation (SD) of coverage per target for each platform. Uniformity is a critical performance parameter in amplicon-based sequencing, as it ensures reliable variant detection across all genomic regions, minimizing the risk of low confidence loci or allelic dropout [29].

Boxplot visualization of per-amplicon SDs demonstrates that Illumina exhibits a tighter distribution, with a narrower interquartile range and fewer high SD outliers, reflecting greater consistency in coverage across the *CFTR* panel. MGI, while achieving higher overall mean coverage, displays slightly increased variability, as evidenced by a broader spread of values and a greater number of extreme outliers.

Importantly, we also evaluated the presence of “critical amplicons,” defined as those with a mean coverage below the 100× diagnostic threshold. No amplicons fell below this threshold on either platform, confirming the robustness of the Devyser CFTR panel and its compatibility with both sequencing technologies. This finding highlights the panel’s reliability in maintaining sufficient coverage depth even in the most challenging regions.

These results align with previously published platform comparisons [23,30], in which Illumina has been shown to provide more uniform read distribution, while MGI offers throughput with a marginal compromise in uniformity. Together, the absence of sub threshold amplicons and the observed performance metrics confirm that both platforms are well suited for high confidence, clinical grade NGS of the *CFTR* gene.

### 2.4. Comparative Analysis of Mean Coverage Across Platforms

To quantitatively assess sequencing depth distribution, we compared the mean amplicon coverage achieved with Illumina and MGI platforms using a boxplot representation. The analysis clearly shows that MGI produces higher mean coverage per amplicon than Illumina, with a median value that substantially exceeds that of its counterpart.

Specifically, the MGI platform displays a wider interquartile range and more frequent high-coverage outliers, suggesting enhanced sequencing efficiency and greater data yield. In contrast, Illumina coverage values are more narrowly distributed, reflecting a tighter but lower average depth.

This higher coverage achieved by MGI can offer benefits in terms of improved detection sensitivity, especially in applications requiring high confidence variant calling or deeper sequencing of low frequency alleles. However, the broader dispersion also reinforces the previously noted trend toward slightly lower coverage uniformity compared to Illumina.

Overall, the results support the notion that MGI offers higher raw performance in terms of sequencing depth, while Illumina maintains consistency and compact coverage distributions features that may appeal to different clinical and research contexts depending on the diagnostic demands.

The comparative evaluation of coverage metrics between Illumina and MGI platforms revealed key performance differences that reflect their respective sequencing architectures. MGI consistently achieved higher mean coverage across all amplicons, as demonstrated through both direct calculations and comparative boxplots. This enhanced depth can enhance analytical sensitivity, particularly in clinical contexts requiring high confidence detection of low frequency variants.

Although MGI exhibited higher overall coverage, the platform showed slightly increased variability across target regions, with a broader spread and higher standard deviations. Illumina, on the other hand, maintained more uniform coverage distribution, despite generating lower read depth. This trait may be beneficial in applications that prioritize coverage homogeneity across all genomic loci.

Importantly, no amplicon on either platform fell below the critical diagnostic threshold of 100×, confirming the robustness of the Devyser CFTR panel across both technologies. Additionally, the relative coverage Δ was consistently positive in favor of MGI, indicating higher sequencing efficiency across the entire panel.

These findings confirm that both platforms are well suited for targeted NGS applications, with MGI favoring throughput and depth, and Illumina excelling in coverage uniformity.

Having established the comparative behavior in terms of coverage, we next examined the sequencing output itself, specifically focusing on the analysis of SNVs, CNVs, and polyT-TG repeat alleles, to assess analytical concordance and variant-level performance across platforms.

### 2.5. Sequencing Quality Comparison—Illumina vs. MGI

To assess the analytical robustness of the two sequencing platforms under investigation, a direct comparison was performed using a standardized Devyser CFTR panel. The results, summarized in Table 1, highlight the most relevant sequencing quality parameters obtained from the MiSeq (Illumina, San Diego, CA, USA) and DNBSEQ-G99RS (MGI Tech Co. Shenzhen, China) systems.

Data Quality, Q30 and Error Rate: The Illumina platform yielded exceptionally high Q30 values, with 95.9% of bases reaching Q ≥ 30 in Read 1 and 91.8% in Read 2. These figures overcome the commonly accepted clinical grade thresholds for base quality and are well aligned with international best practices for diagnostic sequencing. The corresponding Q30 values on MGI were slightly lower (92.75% for Read 1 and 89% for Read 2) but still within the range required for reliable variant detection. Although error rate (0.63%) was reported only for Illumina, the high Q30 metrics achieved by MGI suggest a similarly high base-calling accuracy. Notably, Q30 percentages exceeding 90% are widely regarded as indicative of sequencing reliability in clinical applications [31].

Run Performance: Phasing and prephasing metrics [32], which assess nucleotide incorporation synchrony across reads, were slightly better in Illumina (0.10–0.01% and 0.05–0.01%, respectively) compared to MGI (0.14–0.20% and 0.10–0.10%). Improved phasing metrics are associated with higher sequencing accuracy, particularly in repetitive or GC rich regions. Demultiplexing efficiency was extremely high on both systems, with 96.6% of reads correctly assigned in Illumina and 97.4% in MGI, demonstrating excellent barcoding and sample separation capabilities, crucial for multiplexed diagnostic workflows.

Throughput and Scalability: The total number of filtered reads (PF reads) generated on MGI was approximately 87.8 million, significantly higher than the 5.6 million reads produced by Illumina. This reflects the higher throughput design of the DNBSEQ-G99RS platform (24–48 Gb vs. 1.2 Gb for MiSeqDx), positioning MGI as a strong candidate for high-volume testing, population screening, or other applications where sequencing capacity is a primary concern.

Platform-Specific Metrics: The MGI platform additionally reports internal performance indicators not available in Illumina systems, such as Chip Productivity and Effective Spot Rate (ESR), both measured at 58.58% in this study. While slightly below the commonly cited optimal threshold of 70%, these values still reflect solid run performance and may improve with protocol refinements or updated chemistry. The Recover Value defined as the intensity ratio of Read 2 to Read 1 was 1.50, suggesting a good signal balance and paired-end integrity across the run. Extended QC metrics are referred in Supplementary Materials Table S1.

**Figure 3:**
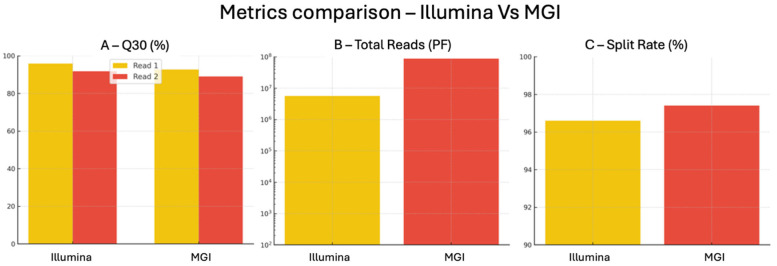
**QC Metrics comparison.** (**A**) **Base Calling Quality** (**Q30 & Error Rate**)**:** This panel compares the quality of sequenced bases across the two platforms, as represented by the Q30 metric defined as the percentage of bases with an error probability ≤ 1 in 1000. Illumina achieved higher Q30 values for both Read 1 (95.9%) and Read 2 (91.8%), indicating excellent base calling performance. MGI also reported strong values (92.75% for Read 1 and 89% for Read 2), which remain fully compatible with diagnostic applications. The error rate was available only for Illumina and was measured at 0.57%, well below the critical 1% threshold, suggesting greater fidelity in base calling, particularly during early sequencing cycles. (**B**) **Phasing and Prephasing:** These metrics reflect the level of synchronization between clusters or DNBs during the sequencing process. Phasing indicates delayed nucleotide incorporation (run-on), while prephasing captures early incorporation (lag). Illumina displayed better performance in both metrics: phasing (0.10% vs. 0.10%) and prephasing (0.05% vs. 0.14%). The improved synchronization observed with Illumina likely contributes to reduced background noise and higher accuracy in sequencing challenging or repetitive genomic regions. (**C**) **Split Rate** (**Demultiplexing Efficiency**)**:** The split rate, or the percentage of reads correctly assigned to their respective samples, was high in both platforms: 96.6% for Illumina and 97.41% for MGI. These values indicate that MGI is well-suited for high-throughput workflows, while Illumina maintains comparable performance in sample demultiplexing for standard clinical applications.

While Illumina provided marginally superior quality scores and synchronization metrics, the MGI platform delivered significantly greater sequencing output, slightly better demultiplexing, and a broader set of performance diagnostics. Both systems met or exceeded key quality benchmarks and demonstrated full compatibility with the targeted *CFTR* assay used in this study. These findings, as resumed in Table 1 and Figure 3, support the analytical equivalence of Illumina and MGI platforms for diagnostic grade sequencing and offer laboratories the flexibility to adopt either platform depending on sample throughput needs, technological infrastructure, and cost-efficiency considerations.

### 2.6. Single Nucleotide Variants

A total of 47 genomic DNA samples were analyzed using a targeted *CFTR* screening approach, which included 371 known pathogenic SNVs small indels and 8 large rearrangements. Among the cohort, both technologies Illumina and MGI, detected clinically relevant SNVs in 6 samples (12.8%), with complete agreement on variant presence and zygosity. The remaining 41 samples were uniformly classified as wild type, yielding a 100% concordance rate between the two platforms in terms of variant detection.

Of the 6 positive cases, five carried variants classified as “CF causing” based on ACMG/AMP guidelines and supporting data from ClinVar and CFTR2 database. One sample harbored a variant of VCC, which was similarly reported by both technologies, emphasizing consistency not only in detection but also in interpretative classification. The observed pathogenic variants spanned exonic regions commonly associated with classical CF phenotypes.

Quantitative analysis of variant allele frequency (VAF) demonstrated platform-specific characteristics. Illumina generated VAF values with low dispersion (mean: 48.3%; standard deviation: 1.78), consistent with the expected profile of heterozygous calls in a targeted amplicon setting. MGI also delivered high-fidelity calls, although with slightly broader variability (mean: 46.8%; SD: 3.36). Despite this range, all VAFs remained within clinically acceptable thresholds for heterozygous interpretation, and no discrepancies in genotype call were observed.

These results confirm that both sequencing platforms are analytically robust and fully interoperable for the detection of clinically relevant *CFTR* mutations screening panel (Table 2). The observed technical reproducibility and interpretative concordance support their interchangeable use in diagnostic pipelines, including applications involving high-throughput or decentralized laboratory networks.

Comparison of detection status, clinical classification, and VAF metrics for the 47—sample cohort analyzed using the Devyser CFTR NGS panel. Perfect concordance was observed between the platforms in both variant identification and clinical interpretation. Illumina showed more consistent VAF values, while MGI exhibited slightly broader variation, though still within diagnostic thresholds for heterozygosity.

This comprehensive analytical approach involved full gene *CFTR* sequencing and extended CNV evaluation, enabling the detection of rare and non-canonical variants across exonic, intronic, and regulatory regions. The goal was to facilitate a more granular and technically rigorous comparison between Illumina and MGI sequencing platforms.

This strategy allowed for direct cross platform evaluation of coverage depth, variant concordance, and platform-specific performance in the context of full gene resolution, thus refining the comparative analysis and expanding the study’s translational significance.

This extended analysis encompassed all 27 exons, exon–intron boundaries and selected intronic regions of known functional relevance (notably introns 7, 9, 11, 12, and 22). The aim was to increase the diagnostic yield by capturing rare, non-hotspot, or deep intronic variants potentially missed by targeted mutation panels.

The same DNA libraries used for the screening were processed using both Illumina and MGI platforms. Variant detection was again performed with the Amplicon Suite software (SmartSeq v3.7.0), with consistent bioinformatic parameters across technologies. A total of 145 unique variants were identified in 35 of the 47 samples (74.5%), all of which were concordantly detected by both platforms.

Quantitative comparison of VAFs further confirmed the analytical agreement between systems. Illumina reads showed VAFs centered around 48–51%, with a standard deviation of 1.78, whereas MGI values ranged from 40.4% to 50.2%, with a SD of 3.36. The maximum absolute VAF difference between the two platforms was <7.5% in all cases, indicating high reproducibility in variant quantification. No false positives or negatives were observed in any sample.

These findings underscore the suitability of both platforms for comprehensive *CFTR* genotyping in a diagnostic setting.

Both Illumina and MGI provided robust, concordant data, supporting their interoperability and diagnostic reliability even in extended genomic contexts.

### 2.7. Copy Number Variants

The CNV screening demonstrated perfect concordance between the Illumina and MGI platforms. All CNV positive regions detected in the dataset were identified consistently by both systems. Specifically, in sample 46, the well-known CFTRdele2,3 deletion was reliably detected by both technologies. The affected amplicons and target regions CFTR_ex02_01, ex02_02, ex03_01, ex03_02, and their respective aggregate target regions CFTR_EX02 and CFTR_EX03 all showed a monoallelic signal (allele count = 1) across platforms, reflecting a clear heterozygous deletion.

While other potential CNV events were flagged by the software in additional samples, they were classified as “unreliable” or low-quality in both datasets and not considered clinically actionable. Importantly, there were no false positives or false negatives for any of the CNV events across the 47 sample.

This level of agreement strongly supports the technical robustness and analytical reliability of both sequencing workflows. Furthermore, it corroborates findings in the literature reporting high CNV calling accuracy using amplicon-based approaches, especially when applied to clinically validated panels like Devyser CFTR [23,29,30]

The ability to correctly identify CFTRdele2,3, one of the most common large deletions associated with classical CF, is of particular diagnostic importance. The concordant detection across both systems reaffirms the clinical applicability of both platforms in first line CFTR genetic testing workflows.

### 2.8. PolyT-TG Screening

The poly-T and TG repeat analysis within the *CFTR* gene was conducted encompassing full allelic and coverage level profiling. Across all samples, the TG repeat typing (TG(9)–TG(12)) showed complete inter platform concordance, underscoring the reliability of both systems in resolving highly polymorphic loci prone to sequencing artifacts.

Allelic frequency comparisons (expressed as VAF%) demonstrated close agreement between platforms, with average values of 48.5% for Illumina and 47.9% for MGI, and no outlier exceeding a ΔVAF of ±7.6%. These values remained well within the range consistent with heterozygous state detection, and highlight the high analytical fidelity of both technologies. Notably, Illumina’s frequency values showed a slightly tighter distribution, which may reflect lower variance in signal processing or basecalling.

Importantly, HGVS nomenclature assignments for detected alleles were perfectly matched across all cases (47/47), indicating not only comparable raw sequencing performance but also consistent downstream bioinformatic interpretation. This reinforces the applicability of both systems in regulated diagnostic contexts where standardized variant reporting is essential.

In terms of read depth, MGI yielded a substantially higher mean coverage (~2793×) compared to Illumina (~1295×), with greater dynamic range but slightly increased variance. This trade-off between depth and uniformity is consistent with previous comparative studies and suggests platform-specific sequencing efficiencies. Despite these differences, both systems exceeded the minimal threshold for confident genotyping and showed no dropout events or coverage gaps across the repeat-containing amplicons.

**Figure 4:**
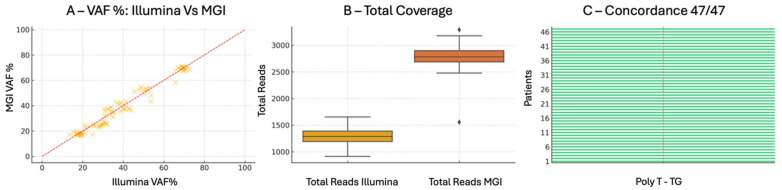
Comparative Analysis: VAF, Coverage, and Concordance. (**A**) VAF% Comparison of Illumina vs. MGI. This panel presents a scatter plot comparing VAF measured on each sample using Illumina and MGI platforms. Each data point represents a sample where at least one variant was identified within the Poly-T/TG region of the CFTR gene. The red dashed line represents the line of perfect agreement (x = y) between the two technologies. Data points are tightly clustered along the diagonal, indicating excellent concordance in allele frequency estimates. No systematic outliers or platform-specific biases are observed, confirming the quantitative accuracy of the library conversion and sequencing process on MGI. (**B**) Total Coverage (Read Depth) per Sample. The second panel shows a comparative boxplot of total raw read counts (coverage) generated per sample on Illumina and MGI. MGI consistently yields higher coverage, consistent with its higher per-run sequencing output (up to 48 Gb). Nonetheless, Illumina also comfortably exceeds the clinical threshold of 100× required per amplicon, ensuring sufficient read depth for accurate allele calling. (**C**) Concordance of c.HGVS Annotations. The final panel displays a binary heatmap assessing concordance of c.HGVS annotations between the two methods across all valid samples. Each row represents a unique sample. All samples are marked in green, indicating complete (100%) concordance in variant annotation between Illumina and MGI. No discordant or missing calls were observed.

Collectively, the findings displayed in Figure 4 support the reproducible performance and interchangeable use of either platform for the analysis of poly-T/TG tracts in *CFTR*, with MGI offering deeper coverage and Illumina providing tighter frequency distributions. These strengths may be leveraged according to clinical priorities, whether focused on maximum read depth or uniformity across target loci.

### 2.9. Turnaround Time (TAT) Analysis and Workflow Comparison

An in-depth assessment of turnaround time (TAT) was conducted to compare the operational workflows of the Illumina MiSeqDx and MGI DNBSEQ-G99RS platforms when processing the same CE-IVD Devyser CFTR panel. Both protocols shared identical upstream phases, including automated DNA extraction, primary multiplex PCR (PCR1), secondary indexing PCR (PCR2), library pooling, magnetic bead-based purification, and Qubit quantification. These steps, executed using standardized automation on the Hamilton STARlet and STAR platforms, required approximately 10 h of hands-on and instrument time.

From this point, procedural differences emerged. The Illumina workflow proceeded directly to denaturation, PhiX spiking, and sequencing setup, culminating in a sequencing run of approximately 18 h (2 × 150 bp), yielding ~1.2 Gb of output per run.

In contrast, MGI required an additional step of library conversion (~5 h), utilizing the Universal Library Conversion Kit (MGI Tech Co. Shenzhen, China) to circularize Illumina-compatible libraries and generate DNBs for patterned flow cell loading. Despite this added complexity, the G99RS platform achieved a significantly faster sequencing time (~12 h), with data output reaching 24–48 Gb per run, substantially higher than Illumina’s.

Illumina workflow, inclusive of extraction, library preparation, pooling, purification, quantification, and sequencing, required approximately 28 h and 15 min to complete, primarily driven by the longer sequencing runtime (~18 h on MiSeqDx). In contrast, the MGI workflow completed the full process in approximately 27 h and 15 min, benefiting from faster sequencing (~12 h on DNBSEQ-G99RS)

However, MGI’s throughput and data volume offer key advantages in high-multiplexing or large cohort settings, while Illumina remains more streamlined due to native compatibility with the library chemistry and fewer protocol adjustments.

**Figure 5:**
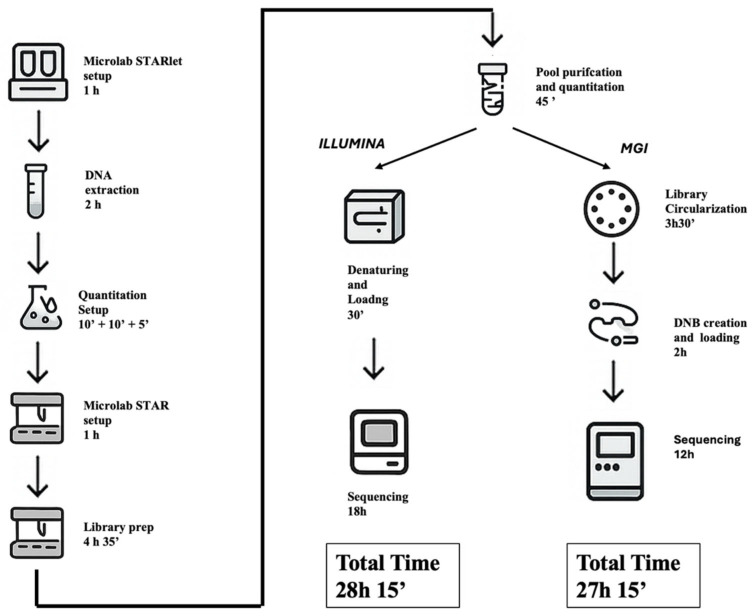
Workflow comparison and TAT for Illumina and MGI sequencing platforms. The diagram outlines the end-to-end laboratory workflow used for *CFTR* gene analysis using the Devyser CFTR NGS kit on Illumina MiSeq and MGI DNBSEQ-G99RS platforms. Shared initial steps include automated DNA extraction, quantification, and library preparation using the Hamilton Microlab STAR system, followed by library pooling and quantification. Downstream, the Illumina workflow includes library denaturation and loading (~30 min) and sequencing (~18 h), leading to a total TAT of 28 h 15 min. The MGI workflow involves additional steps for library circularization (3h 30′), DNBs creation and loading (2 h), and sequencing (~12 h), yielding a slightly reduced total time of 27 h 15 min. Both platforms demonstrated similar end-to-end durations, highlighting their feasibility for timely diagnostic integration.

Ultimately, both systems demonstrated comparable operational timelines and suitability for clinical diagnostics, but their architectural differences, particularly in sequencing chemistry, data output, and required library handling, may inform lab-specific decisions regarding scalability, automation potential, and integration into existing workflows (Figure 5).

## 3. Discussion

The comparative performance analysis of MiSeq (Illumina, CA, USA) and DNBSEQ-G99RS (MGI Tech Co. Shenzhen, China) platforms for *CFTR* gene sequencing reveals that both technologies meet, and in many respects exceed, the rigorous technical demands of clinical grade diagnostics. By evaluating a comprehensive set of quality metrics, ranging from basecalling fidelity and coverage uniformity to variant detection sensitivity and structural variant annotation, this study provides technically consistent evidence for the analytical equivalence and clinical interchangeability of these NGS systems.

From a **data quality standpoint**, Illumina demonstrated slightly higher Q30 scores and lower phasing/prephasing values, reflecting a marginal advantage in read synchronization and basecall precision. Nevertheless, MGI maintained Q30 levels consistently above 89% in both read directions, fully compatible with clinical diagnostic standards. These observations are congruent with previous benchmarking efforts, where both platforms were shown to achieve the base accuracy required for high-confidence variant calling [23,24].

Critically, the **concordance in SNV**, **CNV**, **and complex allele detection** was 100% across 47 independent samples, encompassing both clinically relevant mutations and reference materials with known variant profiles. These include challenging targets such as *CFTRdele2,3* and poly-T/TG tracts, for which both platforms showed full alignment at the HGVS nomenclature level. Such perfect concordance confirms not only the analytical sensitivity and specificity of the platforms, but also the downstream compatibility of bioinformatics pipelines employed (ClinVar, CFTR2, Amplicon Suite v3.7.0) [33,34].

The **coverage analysis** highlighted a significant throughput advantage for MGI, with average read depths exceeding 2700× per amplicon, compared to ~1300× on Illumina. This superior depth, paired with high demultiplexing efficiency (97.41%), underscores MGI’s aptitude for high-throughput diagnostic pipelines, large-scale carrier screening, and population genomics initiatives. Conversely, Illumina demonstrated uniformity and stability of allelic frequencies (VAF%), with tighter interquartile ranges across all heterozygous calls traits that are especially advantageous in low allele fraction detection, mosaicism, or quantitative variant burden analyses.

Moreover, MGI’s proprietary metrics such as **Chip Productivity**, **Effective Spot Rate**, and **Recover Value** offer valuable insights into run efficiency and flow cell performance. Although the observed Chip Productivity (58.6%) was slightly below the nominal optimal, it did not compromise variant detection accuracy or cause amplicon dropout, reinforcing the resilience of the platform even under suboptimal loading conditions. The Recover Value of 1.50 further suggests balanced paired-end signal intensities, a critical parameter for structural variant detection and phasing algorithms [30].

The perfect annotation concordance across **Poly-T/TG alleles** regions known for their sequencing complexity due to low complexity and homopolymeric repeats further validates the capability of both systems to deliver clinically reliable genotyping in non-coding but disease-modifying contexts. This is of particular importance in the interpretation of *CFTR* variants of variable penetrance and their impact on residual function phenotypes.

A comparative assessment of the end-to-end **TAT** for both Illumina and MGI workflows revealed notable differences in sequencing duration and operational logistics, while maintaining overall analytical equivalency. Starting from genomic DNA extracted from 47 clinical samples, both protocols employed the CE-IVD Devyser CFTR NGS kit and followed standardized automated library preparation using the Hamilton Microlab STAR platform. While the total processing time was slightly shorter for MGI, both workflows demonstrated efficient automation and high-throughput capacity, making them suitable for diagnostic settings with varying throughput needs. Importantly, both platforms fit comfortably within a single working day for pre-sequencing steps, with overnight sequencing enabling next day data analysis. These findings support the flexible integration of either platform into clinical laboratory pipelines, depending on infrastructure and scheduling constraints.

From a **clinical implementation perspective**, the results strongly support the idea that both Illumina and MGI can be seamlessly integrated into accredited diagnostic pipelines. Their ability to consistently achieve high-quality metrics across SNV, CNV, and repeat-rich loci confirms their readiness for use in hereditary disease screening, diagnostic confirmation, and pharmacogenetic profiling. This is in line with recent evidence showing how NGS has transformed both germline and somatic diagnostics, extending its role from inherited disease testing to cancer genomics, minimal residual disease monitoring, and liquid biopsy applications [35,36].

Importantly, clinical implementation requires not only accuracy but also adequate coverage and sequencing depth, which are essential for the reliable detection of low-frequency variants in somatic contexts. In applications such as ctDNA analysis, where variant allele frequencies may fall below 1%, only platforms capable of sustaining deep and uniform coverage can ensure analytically reliable detection [37,38]. Our comparative results confirm that both Illumina and MGI surpass the diagnostic coverage threshold, with MGI delivering higher per-amplicon depth, a feature particularly advantageous for oncology workflows and liquid biopsy-based assays.

The decision to adopt either platform can thus be guided by contextual factors, such as throughput requirements, cost-effectiveness, local bioinformatics infrastructure, and long-term scalability, without compromising reliability at the patient level. Illumina systems remain the benchmark for coverage uniformity and mature informatic ecosystems, whereas MGI provides greater throughput and depth, facilitating sensitive detection in high-volume or somatic applications. Ultimately, the analytical equivalence demonstrated across platforms provides laboratories with a unique flexibility to align technological adoption with clinical demand while ensuring compliance with ISO-accredited diagnostic standards.

In conclusion, our data indicate a strong analytical concordance between the Illumina and MGI platforms in the detection of CFTR variants, based on a single-batch, single-run comparative analysis. While the observed technical equivalence is promising, these findings should be interpreted within the limits of our study design, which did not include technical replicates or inter-run variability assessments. Future studies should explore longitudinal performance across multiple sequencing batches, users, and instruments to establish the reproducibility and robustness of each platform under routine diagnostic conditions. This is particularly relevant for high-sensitivity applications such as somatic variant detection and liquid biopsy, where ultra-deep coverage and error suppression are essential for capturing low-frequency alleles with clinical reliability.

In this context, Illumina’s well-established uniformity of coverage and narrower allelic frequency distributions, together with MGI’s higher raw throughput and enhanced per-amplicon depth, may provide complementary strengths that can be strategically leveraged depending on clinical demand. Future studies extending platform comparisons to cfDNA/ctDNA and pharmacogenomic assays will be instrumental in defining their respective advantages in precision oncology and beyond.

In an era of precision diagnostics and cost-conscious healthcare delivery, platform flexibility without sacrificing analytical rigor remains a key requirement. The evidence generated in this study supports both MiSeq and DNBSEQ-G99RS as reliable and interoperable solutions for clinical-grade *CFTR* genotyping, reaffirming that diagnostic robustness and scalability need not be mutually exclusive. By demonstrating platform interchangeability, this work provides laboratories with the freedom to align sequencing strategies with local clinical priorities, ensuring both high-quality patient care and long-term sustainability.

## 4. Materials and Methods

The molecular analysis was conducted at the Genetic and Cytogenetic Unit (UOSD Genetica e Citogenetica) of the Ospedale all’Angelo-ULSS 3 Serenissima, Venice, Italy, a clinical genetics laboratory embedded within the regional reference network for inherited diseases. The study was designed to compare the analytical performance of two high-throughput sequencing technologies, MiSeq (Illumina, San Diego, CA, USA) and DNBSEQ-G99RS (MGI Tech Co. Shenzhen, China), in the targeted sequencing of the *CFTR* gene, using a CE-IVD certified NGS panel developed by Devyser Diagnostics. The experimental workflow adhered to national and international guidelines for *CFTR* variant testing, particularly those outlined in the 2019 Italian Consensus (Società Italiana per lo Studio della Fibrosi Cistica-SIFC, Società Italiana di Andrologia e Medicina della Sessualità-SIAMS, Società Italiana di Biochimica Clinica e Biologia Molecolare Clinica-SIBioC, Società Italiana di Genetica Umana-SIGU) [39].

A total of 47 genomic DNA samples were analyzed, comprising routine clinical cases and archived diagnostic specimens (residual samples), all processed following informed consent procedures in accordance with the EU General Data Protection Regulation (GDPR 2016/679) and Italian privacy laws. The sample set included 45 CF screening cases, 2 external run controls, one harboring a variant of varying clinical consequence (VCC) [40] and one with a known CF causing mutation, and one no template control (NTC) using molecular grade water. The use of residual diagnostic material was approved for analytical validation purposes, and all samples were analyzed in a blinded fashion.

DNA extraction was performed using an automated magnetic bead-based method, specifically the Hamilton Microlab STARlet platform (Hamilton Company, Reno, NV, USA), coupled with the ELITe GALAXY 300 Extraction Kit (ELITech Group S.p.A. Torino, Italy). Elution was carried out in 200 μL volumes, and all samples underwent quantification using the Qubit 4 Fluorometer (Thermo Fisher Scientific, Waltham, MA, USA) with the dsDNA High Sensitivity Assay Kit. Based on the quantification results, DNA concentrations were normalized to 2 ng/μL, using dilution volumes calculated via the standard molarity equation C_1_V_1_ = C_2_V_2_. Samples were then vortexed and stored at −20 °C pending library preparation.

As mentioned above, targeted sequencing libraries were generated using the Devyser CFTR NGS kit, a CE-IVD multiplex PCR-based system designed to detect variants in the *CFTR* gene, including SNVs, indels, and select exon-level CNVs. The kit covers all 27 exons, key regulatory and deep intronic regions (notably introns 7, 9, 11, 12, and 22), and known deletion breakpoints associated with classical and atypical CF phenotypes [41]. Library preparation was fully automated using the Hamilton Microlab STAR liquid handling platform (Hamilton Company, Reno, NV, USA), following a two-step PCR protocol: an initial multiplex amplification (PCR1), followed by sample-specific dual indexing (PCR2) using Devyser’s proprietary plate-based index system. Thermal cycling was performed using On Deck Thermal Cycler (Inheco GmbH, Planegg Germany) instrument with locked ramp rate parameters to ensure reproducibility and amplification efficiency.

Following amplification and indexing, libraries were pooled and subjected to magnetic bead-based purification using the Devyser Library Clean Kit. Post cleanup quantification was repeated by Qubit fluorometry (Thermo Fisher Scientific, Waltham, MA, USA), and the final library pools were diluted to platform-specific concentrations. For Illumina sequencing, pools were adjusted to 0.33–0.41 ng/μL, whereas MGI sequencing required modified dilutions following a conversion step to ensure platform compatibility.

Sequencing was performed on two distinct NGS systems. The first was the Illumina MiSeqDx, configured for paired-end 2 × 150 bp reads using the MiSeq Reagent Kit v2 Micro, which provides up to 1.2 Gb of output. To enhance base diversity during cluster generation, 1% PhiX v3 control (Illumina, San Diego, CA, USA) was included in every run. Library denaturation was achieved using freshly prepared NaOH 0.2N, and run setup was managed through Illumina Experiment Manager, with predefined Sample Sheets ensuring proper index demultiplexing.

The second platform was the MGI DNBSEQ-G99RS, which employs DNB formation and cPAS chemistry. The libraries prepared for Illumina were rendered compatible with MGI’s system using the Universal Library Conversion Kit (App-A) (MGI Tech Co. Shenzhen, China), which facilitates circularization and nanoball generation. Sequencing was conducted with the G99 SM App-C FCL PE150 reagent kit, configured for paired-end 2 × 150 bp reads and delivering an output of 24–48 Gb per run, depending on the flow cell loading density.

Sequencing performance on the MGI platform was evaluated using platform-specific metrics: Chip Productivity (proportion of DNBs successfully bound and producing signal), Total Reads (filtered, usable reads), Q30 score (percentage of bases with a Phred score ≥ 30), Split Rate (index demultiplexing success), Effective Spot Rate (ESR) (spot occupancy), and Recover Value (relative intensity between Read 1 and Read 2). These metrics were used to validate instrument function, data quality, and run integrity, and were compared directly to analogous Illumina metrics such as phasing/prephasing and cluster density.

Raw FASTQ files from both sequencing platforms were processed using Amplicon Suite v3.7.0 (SmartSeq s.r.l. Novara, Italy), a CE-IVD certified software solution for targeted amplicon analysis. The pipeline included adapter and quality trimming (Phred Q < 20), alignment to the GRCh38 human reference genome, and variant calling for both SNVs and indels. CNV detection was performed using normalized depth-of-coverage analysis across predefined *CFTR* regions of interest. Demultiplexing was carried out based on dual index combinations provided by the Devyser platform.

All detected variants were classified according to ACMG/AMP 2015 guidelines [42], using supporting data from public repositories such as ClinVar, CFTR2 database, and gnomAD [43] and The Clinical and Functional TRanslation of CFTR (CFTR2) available at http://cftr2.org, accessed on 20 June 2025 [44]. Pathogenic and likely pathogenic variants were reported as clinically actionable findings. VUS significance were reported with appropriate disclaimers. Quality thresholds included a minimum amplicon coverage of 100×, a Q30 score ≥ 85%, and verification of positive and negative controls. Runs failing to meet these benchmarks were repeated.

## Figures and Tables

**Table 1:** The table provides a head-to-head comparison of the principal sequencing quality metrics generated by the MiSeq platform (Illumina) and the DNBSEQ-G99RS system (MGI) when processing the same NGS panel (Devyser CFTR). This parallel evaluation aims to rigorously assess the analytical performance and reliability of each platform in a standardized clinical context.

Parameter	Illumina	MGI
Total Reads (PF)	5,640,738	87,800,000
Q30 Read 1 (%)	95.9	92.8
Q30 Read 2 (%)	91.8	89.0
Error Rate (%)	0.63	n/d
Phasing Read 1 (%)	0.10	0.14
Phasing Read 2 (%)	0.01	0.20
Prephasing Read 1 (%)	0.05	0.10
Prephasing Read 2 (%)	0.01	0.10
Cluster PF (%)	79.8	n/a
Split Rate/% PF Identified	96.6	97.4
Chip Productivity (%)	n/a	58.58
Effective Spot Rate (ESR)	n/a	58.58
Recover Value (R2/R1)	n/a	1.50

**Table 2:** Summary of SNV Screening and Concordance Between Illumina and MGI Platforms.

Category	Metric	Illumina	MGI
Detection Status	Detected	6	6
	Not detected	41	41
Variant Classification	CF-causing	5	5
	VCC	1	1
VAF Distribution	Mean VAF (%)	48.3%	46.8%
	Min–Max VAF	46.3–51.6	40.4–50.2
	Standard Deviation	1.78	3.36

## Data Availability

The original contributions presented in this study are included in the article/Supplementary Material. Further inquiries can be directed to the corresponding author.

## Supplementary Materials

The following supporting information can be downloaded at https://www.mdpi.com/article/10.3390/ijms262311701/s1.
