# Supplementary material for: Methodological Assessment of High-Throughput Sequencing Platforms: Illumina vs. MGI in Clinical-Grade CFTR Genotyping
Source: Int J Mol Sci. 2025 Dec 3;26(23):11701. doi: 10.3390/ijms262311701 (PMC12692382; doi:10.3390/ijms262311701)
Supplement: Supplementary file 1 [file ijms-26-11701-s001.zip › ijms-4023177-supplementary.pdf]

Supplementary QC Metrics, Technical Overview and Platform-Specific Considerations

To further substantiate the analytical robustness of the comparative study, extended QC metrics were extracted from native sequencing report files, including Illumina's Sequencing Analysis Viewer (SAV) outputs and MGI's run summary. These data expand upon the core quality indicators described in the main manuscript by providing greater transparency and technical granularity.

On the Illumina MiSeq platform, the cluster density was reported at 1203 ± 34 K/mm², with Cluster PF rate of 79.81 ± 2.54%, aligning with manufacturer specifications. Phasing and prephasing values for Read 1 remained low (0.10% and 0.05%, respectively), indicating minimal signal decay or synchronization loss across the 300-cycle paired-end run. Notably, the Q-score heatmap demonstrated homogeneous quality across the flow cell surface with isolated declines corresponding to adapter trimming boundaries. The Q30 rate for Read 1 was 95.9%, and average alignment rates exceeded 97.5%, with an average error rate per cycle under 0.7%. The index-hopping estimate, indirectly assessed through the %PF Identified values and CV across indices, showed limited dispersion (CV ≈ 0.17%), confirming minimal barcode bleeding across samples.

For the MGI DNBSEQ-G99RS platform, chip productivity was recorded at 58.58%, with an Effective Spot Rate (ESR) also at 58.58%, reflecting the consistency of usable DNBs. Recover Value (R2/R1) reached 1.50, consistent with dual-index sequencing stability. Although base-by-cycle phasing metrics are not directly reported in the summary, the alignment rates remained high (>98%), and the split rate (PF Identified %) was 97.41%, with well-distributed index recovery. Signal intensity and cumulative Q-score profiles showed consistent performance across tiles and cycles.

Overall, the supplementary metrics reinforce the platform-level equivalence observed in variant detection, with each system exhibiting reliable sequencing behavior and quality output. While Illumina showed slightly superior per-cycle Q-scores and lower phasing, MGI compensated with increased read volume and efficient demultiplexing. These extended metrics provide transparency for reproducibility assessments and enable readers to independently evaluate the technical robustness of the dataset beyond variant concordance alone.

Metric	Illumina	MGI
Read Yield (Gbp)	1.59 Gbp	13.3 Gbp
Aligned Reads (%)	98.2%	97.5%
Average Coverage Depth (X)	932x	2793x
Minimum Coverage Depth (X)	319x	1450x
Maximum Coverage Depth (X)	2358x	3902x
Amplicon Dropout Events	None	None

Index Hopping Observed	None	None
NTC Contamination Detected	No	No
QScore Drift Observed (R1)	Negligible	Stable
QScore Drift Observed (R2)	Negligible	Stable

Supplementary Table S1: Extended QC Metrics of Illumina and MGI Platforms. This table reports additional sequencing quality metrics obtained from raw run files (Illumina SAV and MGI summary). It includes cluster density, aligned reads percentage, error rates per cycle, index representation uniformity, and Q-score heatmap distribution. These metrics provide further insight into platform-specific performance in terms of signal intensity, spatial flowcell behavior, sequencing fidelity, and sample demultiplexing, thereby supporting a more granular technical comparison beyond conventional metrics.
